# Influence of Directed Energy Deposition Build Orientation on the Final Mechanical Properties of Large Ti-6Al-4V Components

**DOI:** 10.3390/ma19194142

**Published:** 2026-09-28

**Authors:** Luca Marsilio, Lorenzo Pollicini, Federico Mazzucato, Mohammad Taghian, Abdollah Saboori, Anna Valente

**Affiliations:** 1 Automation, Robotics and Machines Laboratory, Department of Innovative Technologies (DTI), University of Applied Science of Southern Switzerland (SUPSI), 6962 Lugano-Viganello, Switzerland; luca.marsilio@supsi.ch (L.M.); lorenzo.pollicini@supsi.ch (L.P.); anna.valente@supsi.ch (A.V.); 2 Department of Management and Production Engineering (DIGEP), Politecnico di Torino, 10129 Turin, Italy; mohammad.taghian@polito.it (M.T.); abdollah.saboori@polito.it (A.S.); 3 Integrated Additive Manufacturing Center (IAM@PoliTo), Politecnico di Torino, 10129 Turin, Italy

**Keywords:** additive manufacturing, directed energy deposition, oxygen pick-up, build rates, mechanical properties

## Abstract

Directed Energy Deposition (DED) is an additive manufacturing technology rapidly consolidating for the fabrication and repair of complex aerospace Ti-6Al-4V components, ensuring high deposition rates and large build volumes. However, in high build-rate regimes, process-induced phenomena, such as oxygen pick-up, overheating, and residual porosity, introduce material anisotropy. While residual porosity can largely be mitigated by Hot Isostatic Pressing (HIP), dissolved oxygen cannot be removed by any post-processing method, raising uncertainties regarding the final mechanical properties of large DED parts and limiting the predictive performance of design simulation tools. To fully exploit the advantage of design for additive manufacturing methods, it is fundamental to assess how the DED process influences the material performance of large Ti-6Al-4V builds. This work investigates the effect of build orientation and process parameter set on the final mechanical behaviour of DED Ti-6Al-4V samples in high-build-rate conditions. Compared to cast and annealed Ti-6Al-4V, all the realized samples exhibit an average 14% and 22.5% increase in yield strength and ultimate tensile strength, respectively, but 38% lower Elongation at Break. Microstructural analysis reveals that both the process parameter set and the build orientation influence DED material anisotropy, particularly ductility, with vertically built specimens showing 68% and 33% higher elongation at break than longitudinal and lateral specimens, respectively, highlighting a direction-dependent mechanical response consistent with the columnar prior β-grain morphology and extension. LECO analysis confirms that oxygen pick-up also occurs in an enclosed deposition environment and strongly increases material strength at the expense of ductility. Oxygen content varies with build orientation, from 0.15 ± 0.02 wt.% in vertical to 0.19 ± 0.01 wt.% in longitudinal specimens. Finally, fracture surface analysis indicates that lack-of-fusion defects act as preferential crack initiation sites.

## 1. Introduction

Titanium grade 5 (also known as Ti-6Al-4V) is an α-β titanium alloy that finds application in relevant industrial sectors thanks to its excellent specific strength (e.g., Aerospace), good biocompatibility (e.g., MedTech), and enhanced corrosion properties (e.g., Maritime) [1]. Its mechanical properties strongly depend on the microstructural distribution and morphology of the α and β phases, which are in turn influenced by the processing methods [2,3,4,5]. For instance, Shaikh et al. observed the formation of martensitic structures consisting of α’ and transformed β for water-quenched Ti-6Al-4V components, which increased Ti-6Al-4V hardness compared to furnace-cooled ones [2]. On the contrary, low cooling rates were demonstrated to increase the content of globular and lamellar α phases instead of secondary α, inducing improved ductility in conjunction with a lower Ultimate Tensile Strength (UTS) [6].

Recently, Additive Manufacturing (AM) technologies gained significant attention in industry for the cost-effective manufacturing of geometrically complex Ti-6Al-4V components, enabling optimized designs and improved functionality compared to conventional manufacturing methods [7]. Among AM techniques, Directed Energy Deposition (DED) is a promising AM technology that finds increasing applications in the Aerospace sector for the manufacturing and repair of high buy-to-fly ratio components [8,9], thanks to its capability to both realize complex Ti-6Al-4V parts [10] and enable local surface functionalization, ensuring high build rates and low material waste. The high cooling rates experienced during the DED process (e.g., typically ranging between 10^2^ K/2 and 10^4^ K/s [11]) affect the final microstructure of Ti-6Al-4V components by conditioning the formation and the distribution of α and β phases and impacting the mechanical strength, ductility, hardness, and fatigue resistance of as-built DED parts [4,12]. Recent studies proved DED melt pool solidification rates can be varied to optimize α and β phase formation by controlling laser energy densities and deposition strategies involved in the process [13,14]. Nursyifaulkhair et al. observed an increase in the α massive phase against α’ by decreasing melt pool cooling rates, detecting the maximum volume fraction at 4344 Ks^−1^ [15]. Bian et al. demonstrated that coarser microstructures are achieved at lower cooling rates by decreasing the traverse speed and increasing the laser power [16]. Due to the rapid solidification process, DED Ti-6Al-4V parts exhibit a finer microstructure than cast parts [17], resulting in higher Yield Stress (YS) and Ultimate Tensile Strength (UTS) [4,11]. According to [18], typical cast YS and UTS values are 885 MPa and 930 MPa, respectively, with 12% elongation. On the contrary, Yu Jun et al. reached 950 MPa YS, 1025 MPa UTS, and 4.9% elongation [4,15,16,17,18].

Together with the distribution and morphology of α and β phases, in DED-fabricated Ti-6Al-4V parts, the resulting mechanical properties are also strongly affected by the directional thermal fluxes that form during the deposition process, which promote the preferential growth of prior β grains along the building direction, following the local thermal gradients established during deposition [14]. This solidification-driven mechanism results in a microstructural anisotropy, leading to a material’s mechanical behaviour that is strongly orientation-dependent. Post-processing (such as solution treatment) is usually performed to homogenize the as-built microstructure and reduce anisotropy. Nevertheless, for complex AM geometries, variations in local cross-section alter the thermal history and lead to heterogeneous microstructures within the same part [19]. While sub-transus annealing can reduce the resulting anisotropy, complete homogenisation generally requires dedicated multi-step or supra-transus treatments [20], which are not always compatible with the dimensional stability requirements of large components.

Besides α-β phases and grain orientation, another significant factor affecting the mechanical properties of DED Ti-6Al-4V components is the absorption and diffusion of interstitial impurities during part manufacturing, such as oxygen [21,22,23,24,25,26]. The influence of oxygen pick-up on the final mechanical properties of Ti-6Al-4V lattice structures produced by the Laser Powder Bed Fusion (L-PBF) process was analysed by Velasco-Castro et al. [27]. The authors observed lower compression resistance in the produced samples due to oxygen-induced material embrittlement. On the contrary, Ter Haar et al. observed a decrease in ductility, accompanied by increases in strength and hardness, with increasing oxygen content [28]. In DED, oxygen pick-up strongly depends on melt pool temperature and cooling rates, which in turn depend on process parameters, build orientation, shielding efficiency, and deposition strategy. As a result, oxygen-related strengthening is unevenly distributed within the fabricated parts, contributing to the anisotropy in the mechanical response of AMed titanium alloys. Moreover, unlike α-β phases and microstructure grain morphology, the absorbed oxygen is hardly removable through post-processing. Carrozza et al. demonstrated that high oxygen contents cause changes in the DED Ti-6Al-4V microstructure, inducing a brittle behaviour of the as-built part [29], while the use of an inert deposition chamber limits but does not prevent oxygen absorption in as-deposited Ti-6Al-4V components [30]. Despite these findings, the analysis was performed on small-sized samples, without executing quasi-static tensile testing. Yang et al. reported a significant effect of oxygen content on the mechanical properties of Ti-6Al-4V [31]. Specifically, when the oxygen content increases to 0.11 wt.%, the UTS increases from 1006 MPa to 1234 MPa, while the elongation to failure markedly decreases from 18.6% to 6.5%, highlighting the strong strengthening effect of oxygen, accompanied by a reduction in ductility. Despite the authors highlighting the detrimental effect of oxygen pick-up during deposition, the influence of building orientation on oxygen absorption was not analysed. The aforementioned studies primarily focus on small-scale specimens, which offer limited applicability at the industrial level, where components can reach dimensions of several cm^3^. Moreover, due to the inherent variability of DED processes, experimental results obtained from small-scale samples are poorly transferable to medium-to large-sized components. From an operational perspective, this intrinsic and irreversible anisotropy poses a significant challenge for the design and qualification of DED-fabricated components, limiting the reliability and accuracy of conventional mechanical simulation tools and predictive performance models, which typically assume homogeneous and isotropic material properties.

Despite the extensive literature on DED Ti-6Al-4V, two main issues remain unresolved: (i) Ti-based alloy anisotropy and the corresponding mechanical properties have been established almost exclusively on small-scale specimens, whose thermal history is not representative of the bulk deposits used in industrial practice, and (ii) the influence of build orientation on oxygen pick-up in medium-to-large-sized components and the resulting mechanical properties has not been quantified yet. Such open points represent a scalability gap that, if left unaddressed, poses a critical challenge to the wider adoption of DED in industrial sectors. For this reason, the scientific community needs to place greater focus on research work that replicates DED manufacturing conditions for medium-to-large-sized components, in order to understand how printing conditions and process parameters influence the final quality of industry-relevant parts.

This research aims to characterize the influence of building orientation and parameter sets on the final mechanical behaviour of medium- to large-scale DED Ti-6Al-4V samples by linking the resulting microstructure and oxygen absorption with the mechanical response observed in quasi-static tensile testing. To this end, Ti-6Al-4V cuboids of industrially relevant size were deposited in three build orientations with two process parameter sets, and the resulting microstructure, oxygen content, tensile response and fracture behaviour were analysed jointly. The expected outcome is a quantitative description of the orientation-dependent property envelope that can be used as input for the design and qualification of DED components.

## 2. Materials and Methods

The material used in this study is a spherical Ti-6Al-4V powder supplied by Powder Alloy Corporation (see Figure 1), with a low satellite count and a particle size distribution between 45 and 105 µm. Table 1 shows the chemical composition of the powder. The reported oxygen value represents the maximum content specified by the powder supplier and not a LECO measurement of the actual powder batch.

All samples are fabricated on 150 × 80 × 10 mm^3^ (X, Y, Z) Ti-6Al-4V substrates by using a homemade 5-axis DED machine designed and developed by the Automation, Robotics and Machines (ARM) research group at SUPSI (see Figure 2a). The system features an 800 × 800 × 1000 mm^3^ (X, Y, Z) building volume and an enclosed deposition chamber enabling the processing of reactive alloys by filling the deposition area with Argon 4.6 and maintaining an oxygen content below 700 ppm during deposition. The actual oxygen content inside the deposition volume is measured before the process starts and during the deposition through a Tecnosens TEC-ZRC 3.0 module based on zirconium oxide sensor. The sensor is installed at the height of the first deposition layer but at a distance sufficient to prevent any damage caused by metal powder and sparks. The deposition head is a multi-nozzle solution featuring four nozzles with 1 mm outlet diameter and a central shielding gas flow that enhances melt-pool shielding and prevents optical contamination from fumes and spatter (see Figure 2b). A uniform and continuous powder supply to the deposition head is ensured by a Flame Spray Technologies powder feeding system. The laser source operates in continuous-wave mode, delivering 1 kW of power at 1070 nm. The beam is delivered through a 600 µm core optical fiber and focused to a 1 mm spot diameter on the substrate.

The samples used in this study are Ti-6Al-4V cuboids, fabricated with three different build orientations and two levels of laser power, for a total of six cuboids. Three samples are produced at a time. For each parameter set, the three cuboids (vertical, lateral, and longitudinal) are deposited within the same job, alternating layer by layer between the three builds. No dwell time was programmed between successive layers (idle time = 0 s in Table 2); the effective interlayer time of each cuboid therefore corresponds to the time required to deposit the corresponding layers of the other builds. Owing to their different heights, the longitudinal and lateral cuboids were completed before the vertical one, after which the vertical cuboid was deposited alone, with no interruption between successive layers. Figure 3a shows the cuboid size and printing orientations, while the levels of laser power (P) and axis speed (F) used in the experimental campaign are reported in Table 2. The selected process parameter sets (P3F9 and P4F9) are based on a previous optimisation study [29,30], in which near-full density was achieved. The cross-section analysis performed through a digital microscope revealed final densities of 99.98% and 99.99%, characterized by randomly distributed porosity with an average pore size of 30 μm. In both sets, hatch distance (h.d.) and layer thickness (dz) are not selected independently of the laser power level. Indeed, a constant 60% single-track overlap and a dz equal to the single-layer height measured for the corresponding power and feed rate combination are imposed. Since both the width and the height of the single-track increase with laser power, the h.d. and dz differ between the two sets by +15% and +26%, respectively (see Table 2). As a consequence, the volumetric energy density, defined as VED = P/(F·h·dz), is 218 J mm^−3^ for P3F9 and 201 J mm^−3^ for P4F9. Despite the 33% higher laser power, the VED of P4F9 is approximately 8% lower. For this reason, the two conditions are hereafter referred to as process parameter sets rather than as laser power levels, and the observed differences are attributed to their combined effect rather than to laser power alone.

After cuboid depositions, residual stresses are relieved in a Carbolite Gero GPCMA/117 (Carbolite Gero GmbH, Neuhausen, Germany) electrical furnace by heating to 600 °C at 5 °C/min, holding at this temperature for 120 min, and then slowly cooling in the furnace for 12 h [32]. The heat treatment is performed in an Argon-filled chamber, keeping an O_2_ content below 0.005% for the entire duration of the heat treatment.

For each cuboid, five tensile specimens are cut through wire Electrical Discharge Machining (EDM) (see Figure 3b). Since the oxygen diffusion coefficient in Ti-6Al-4V is approximately D = 4.1 × 10^−17^ m^2^ s^−1^ at 600 °C [33] and the tensile specimens are extracted from the interior of the deposited cuboids after removing at least 1.5 mm from all external surfaces, any surface-related oxygen contamination introduced during the heat treatment can be excluded. Quasi-static tensile tests are performed in accordance with ASTM E8 using an MTS Alliance RT/100 (MTS Systems Corporation, Eden Prairie, MN, USA) universal testing machine. The tensile deformation was measured using an extensometer. The extensometer had an initial gauge length of 2.5 mm. The tests were performed at a constant crosshead speed of 2 mm/min, corresponding to a nominal strain rate of approximately 0.0133 s^−1^ (0.8 min^−1^), calculated from the crosshead speed divided by the initial gauge length. Therefore, the strain data were obtained directly from the extensometer, providing a more accurate measurement of the specimen deformation than that based on crosshead displacement. Three tensile specimens are tested for each combination of parameters and printing orientation, for a total of 18 tensile tests performed. The effects of build orientation and process parameter set on YS, UTS and E.B. were assessed by two-way analysis of variance (ANOVA) including the interaction term, based on three tensile tests per condition.

After the mechanical tests, microstructural analyses are conducted using a Leica inverted optical microscope (Leica Microsystems GmbH, Wetzlar, Germany). Microstructural observations were performed on a longitudinal section located within the grip region of each tensile specimen. The analyzed section was parallel to the tensile loading axis and included the build direction, allowing the prior β-grain morphology to be evaluated relative to both directions. Because the grip region did not undergo plastic deformation during tensile testing, the observed microstructure was considered representative of the as-built condition.

The samples are mounted, ground, polished, and etched using Kroll’s agent (H_2_O/HF/HNO_3_ = 1:3:50 mL) for 10 s to reveal the microstructure. The fracture surfaces are ultrasonically cleaned in isopropyl alcohol (IPA) and water for approximately 5 min each and subsequently analyzed by Scanning Electron Microscopy (SEM) using a Zeiss EVO50 microscope (ZEISS, Oberkochen, Germany) with a thermionic source to investigate the fracture mechanisms.

Dimple-size measurements were performed on higher-magnification SEM images using ImageJ (v1.54g). Each image was calibrated using its 3 µm scale bar, and the maximum Feret diameter of 20 clearly resolved dimples was manually measured on one representative fracture surface for each processing condition. Dimples intersecting the image boundaries or exhibiting indistinct edges were excluded.

To assess the relationship between oxygen content and the resulting mechanical behavior, the oxygen concentration in the deposited specimens was quantified using a LECO ONH836 analyzer (LECO Corporation, St. Joseph, Michigan, MI, USA) on samples extracted from specimens fabricated with different build orientations.

## 3. Results and Discussion

### 3.1. Microstructural and Mechanical Characterization

Figure 4 shows the microstructure of Ti-6Al-4V tensile specimens fabricated along the longitudinal build direction. The microstructure is dominated by coarse, columnar prior β-grains elongated along the build direction, reflecting the thermal gradients inherent to the DED process. This grain alignment is expected to induce anisotropic mechanical behavior. Within the prior β-grains, a basket-weave microstructure consisting of fine α-laths interwoven with retained β-ribs is observed. Additionally, an acicular morphology is observed, which may be associated with the relatively high cooling rates experienced during deposition compared with those in conventional casting. While these microstructural features contribute to enhanced strength, they also adversely affect ductility.

The influence of these microstructural characteristics is reflected in the quasi-static tensile results reported in Figure 5 and Table 3, which demonstrate a dependence of mechanical properties on parameter sets and build orientation. Pareto charts (see Figure 6) and main-effect plots (see Figure 7) confirm that both the chosen process sets and build orientation have a high impact on the resulting mechanical properties, with ANOVA *p*-values below 0.05. On the contrary, the interaction between P and build orientation results to be negligible for UTS and E.B., but significant for YS, even if its effect is attenuated (*p*-value = 0.0009), if compared to P (*p*-value = 6.3 × 10^−7^) and build orientation (*p*-value = 0.0001). In particular, vertically oriented P4F9 specimens exhibit an average YS of 890 ± 6 MPa, i.e., 11.4% lower than laterally oriented P4F9 specimens (1004 ± 8 MPa), observing statistical significance. A similar orientation-dependent trend is observed for the UTS. The largest effect of build orientation is observed in the elongation at break (E.B.). Vertically built specimens exhibit higher ductility, with elongation approximately 68% higher than that of longitudinal specimens and 33% higher than that of lateral specimens. This orientation-dependent response may be associated with the alignment of elongated prior β-grains relative to the tensile loading direction. However, because strain-hardening behavior, crystallographic texture, and uniform elongation were not evaluated, the underlying deformation mechanisms cannot be conclusively identified.

Compared to standard cast and annealed Ti-6Al-4V Grade 5, the DED-fabricated samples exhibit enhanced tensile strength at the expense of ductility. This combination of properties can be explained by the presence of coarse columnar β-grains coupled with strengthening microstructural constituents such as fine α-laths and a fine acicular morphology. In addition to the build orientations, the tensile results also reveal a measurable influence of the process parameter combinations. P3F9 specimens reach UTS values up to 1200 MPa in the lateral orientation, whereas P4F9 specimens reach a maximum of 1128 MPa, also in the lateral orientation. Overall, the mean UTS of P4F9 specimens (1108 MPa) is approximately 5.3% lower than that of P3F9 specimens (1171 MPa). This difference is statistically significant (*p* = 0.0007) and consistent across the three build orientations. For all investigated orientations, P3F9 specimens consistently display higher strength than P4F9 specimens. In contrast, elongation at break shows the opposite trend, with specimens fabricated at the P4F9 condition generally exhibiting higher ductility. This behavior is likely related to differences in the thermal history experienced during deposition, which may affect cooling rates, phase fractions, and residual stresses.

### 3.2. Fracture Surface Analysis

SEM analysis confirms that the tensile specimens exhibit a mixed fracture mode, combining both ductile and brittle features (see Figure 8a). The central region of the fracture surface is dominated by ductile fracture, evidenced by extensive plastic deformation and the presence of microvoid coalescence and ductile dimples. A noticeable variation in dimple size is observed across the fracture surface, indicating non-uniform local deformation mechanisms.

An increase in dimple size was observed for the P4F9 condition, as shown in Figure 8b,c. Based on the maximum Feret diameter of 20 clearly resolved dimples, the average dimple size was approximately 2.7 ± 0.8 µm for P3F9 and 6.4 ± 1.9 µm for P4F9.

This trend is consistent with the higher ductility observed for the P4F9 samples. It suggests that the modified thermal conditions at P4F9 condition, specifically, increased melt pool volume and reduced cooling rates, promote more void growth prior to coalescence.

The presence of larger dimples and deeper cavity-like features in these samples further supports the occurrence of more extensive plastic deformation before final fracture. In contrast, the peripheral regions of the fracture surfaces exhibit features typical of brittle failure, including cleavage-like facets and limited plastic deformation, indicative of abrupt crack propagation. The coexistence of ductile and brittle zones within the same specimen is characteristic of Ti-6Al-4V under tensile loading and reflects the heterogeneous stress distribution and microstructural gradients produced during the DED process.

Fracture surface observations also reveal the presence of lack-of-fusion (LoF) defects, which are more prevalent in longitudinally built specimens compared to vertically built ones (see Figure 9), agreeing with the generally higher elongation at break measured for vertical specimens. This trend can be explained by the deposition strategy in the DED process.

Longitudinal builds require the fabrication of extended layers, which increases the likelihood of incomplete fusion between tracks due to the limited number of remelting passes along long deposition paths. In contrast, vertically built specimens have smaller layer cross-sections, allowing higher thermal gradients, more complete fusion, and fewer LoF defects.

The effect of build orientation on LoF prevalence is more pronounced for the P4F9 set. It may appear counterintuitive that the higher-power condition exhibits a larger number of LoF defects, since a higher energy input generally promotes complete fusion. However, as reported in Table 2, the volumetric energy density of P4F9 is approximately 8% lower than that of P3F9. Indeed, the 33% increase in P is more than counterbalanced by the 26% larger dz and the 15% wider h.d. The resulting reduction in the ratio between melt pool depth and layer increment limits the remelting of previously deposited material which, combined with the long deposition paths required by longitudinal builds, favours incomplete fusion between adjacent tracks, whereas vertical specimens remain less affected due to their smaller layer dimensions. Consequently, the interplay between build orientation and process parameters contributes to the observed anisotropy in E.B., with longitudinal specimens exhibiting lower ductility relative to vertical ones. It is noteworthy that the assessment of LoF defects presented here is qualitative and based on fracture surface observations. A quantitative defect analysis is required to establish the correlation on a statistical basis, but it was beyond the scope of this work.

### 3.3. Oxygen Content Analysis

Oxygen content in the Ti-6Al-4V specimens was analyzed to assess its influence on the observed mechanical behavior. Oxygen is a well-known interstitial element that acts as a solid solution strengthener in titanium alloys, directly affecting both strength and ductility. In this work, longitudinally built specimens exhibit higher oxygen content (0.19 ± 0.01 wt.%) compared to vertically built samples (0.15 ± 0.02 wt.%) (see Figure 10).

This trend is consistent with the quasi-static tensile results, in which longitudinally oriented specimens showed higher yield and ultimate tensile strength, whereas vertically oriented samples showed greater elongation at break. The higher oxygen uptake observed in longitudinal specimens can be attributed to the longer exposure of the melt pool to the surrounding atmosphere during deposition, as well as the deposition strategy and the kinematics of the DED print head. In specimens fabricated in non-vertical orientations, such as longitudinal or lateral builds, the deposition of extended layers requires wider and more complex movements of the print head across the substrate. As a result, the shielding gas flow, which is locally delivered through the deposition head, may be less uniformly maintained over the entire melt pool area during printing. Conversely, vertically built specimens are characterized by a significantly smaller deposition cross-section, which requires more localized and repetitive deposition passes. In this configuration, the print head remains spatially closer to the melt pool, allowing a more stable and effective coverage of the molten material by the inert shielding gas. This reduced dispersion of the protective gas is expected to limit oxygen ingress during deposition, thereby reducing oxygen pick-up. Since the oxygen diffusion length in Ti-6Al-4V at 600 °C is approximately 0.5 µm over the 120 min holding time (D ≈ 4.1 × 10^−17^ m^2^ s^−1^ [33]), and the tensile specimens were extracted from the interior of the deposited cuboids after removing at least 1.5 mm from all external surfaces, any surface-related oxygen contamination introduced during the heat treatment can be excluded, suggesting that the observed differences in oxygen content are therefore more likely to be introduced during deposition than during subsequent thermal treatment. Similarly, the higher strength and lower elongation at break of the P3F9 specimens might also be partly related to oxygen uptake, since the total deposition time of the P3F9 job was longer than that of P4F9 (118 vs. 93 min), resulting in a longer overall exposure of the deposited material to the process atmosphere. These findings highlight the critical importance of carefully controlling powder handling, shielding efficiency, and deposition strategy during DED processing to minimize interstitial contamination and achieve consistent mechanical performance.

## 4. Conclusions

This work aimed to assess how process parameters and build orientations influence the mechanical performance and microstructural features of Ti-6Al-4V samples produced via DED in high-build-rate conditions. Two process parameter sets, differing in laser power, layer thickness, and hatch distance, were employed for sample fabrication, and the resulting mechanical behaviour and microstructure were systematically analysed.

The achieved findings can be summarized as follows:

1. Microstructural analysis revealed elongated lamellar β-grains aligned parallel to the build direction, leading to direction-dependent mechanical response. The observed fine acicular morphology may contribute to increased strength and reduced ductility, resulting in UTS values exceeding 1180 MPa, higher than those typically reported for cast Ti-6Al-4V.
2. A mechanical anisotropy was observed. Vertically built specimens exhibited an average yield strength reduction of about 11.4% compared to laterally oriented samples, while showing significantly higher elongation at break, with values approximately 68% higher than longitudinal specimens and about 33% higher than lateral ones. This behavior may be associated with the alignment of elongated prior β-grains along the build direction and to orientation-dependent defect distribution. For structural applications, the observed findings highlight the importance of accounting for build orientation in the development and optimization of part design tools with the aim of engineering high-performance DED-manufactured structures.
3. Fracture surface analysis revealed a mixed ductile-brittle behaviour. Central regions displayed ductile dimples and extensive microvoid coalescence, while peripheral areas exhibited cleavage-like facets, indicating brittle crack propagation. The average dimple size increased from approximately 2.7 ± 0.8 µm for P3F9 to 6.4 ± 1.9 µm for P4F9.
4. Lack-of-fusion (LoF) defects were more prevalent in longitudinally built specimens, particularly for the P4F9 set, where they acted as preferential crack-initiation sites. The interaction between build orientation and process parameters significantly contributed to the observed anisotropy in elongation at break and to the overall reduction in mechanical performance. From a process optimization point of view, these results underscore the need to tailor laser power and layer thickness settings to specific build orientations, rather than applying uniform parameters across the entire build strategy.
5. Oxygen absorption varied with build orientation, with longitudinal samples exhibiting higher oxygen pick-up than vertical ones. This trend correlates with increased strength and reduced ductility, consistent with oxygen acting as a solid-solution strengthener in Ti-6Al-4V. Monitoring and controlling oxygen uptake during DED processing emerges as a critical process control variable, particularly for applications where ductility and toughness are primary requirements. These findings highlight the need to implement inert build chambers ensuring ambient oxygen levels below 700 ppm, and to control powder handling and shielding efficiency throughout deposition.

Furthermore, the improved understanding of the relationships between process parameters, build orientation, oxygen uptake, and mechanical response supports the development of more accurate material models for numerical simulations, contributing to enhanced predictive capabilities in the design of DED-fabricated components.

## Figures and Tables

**Figure 1:**
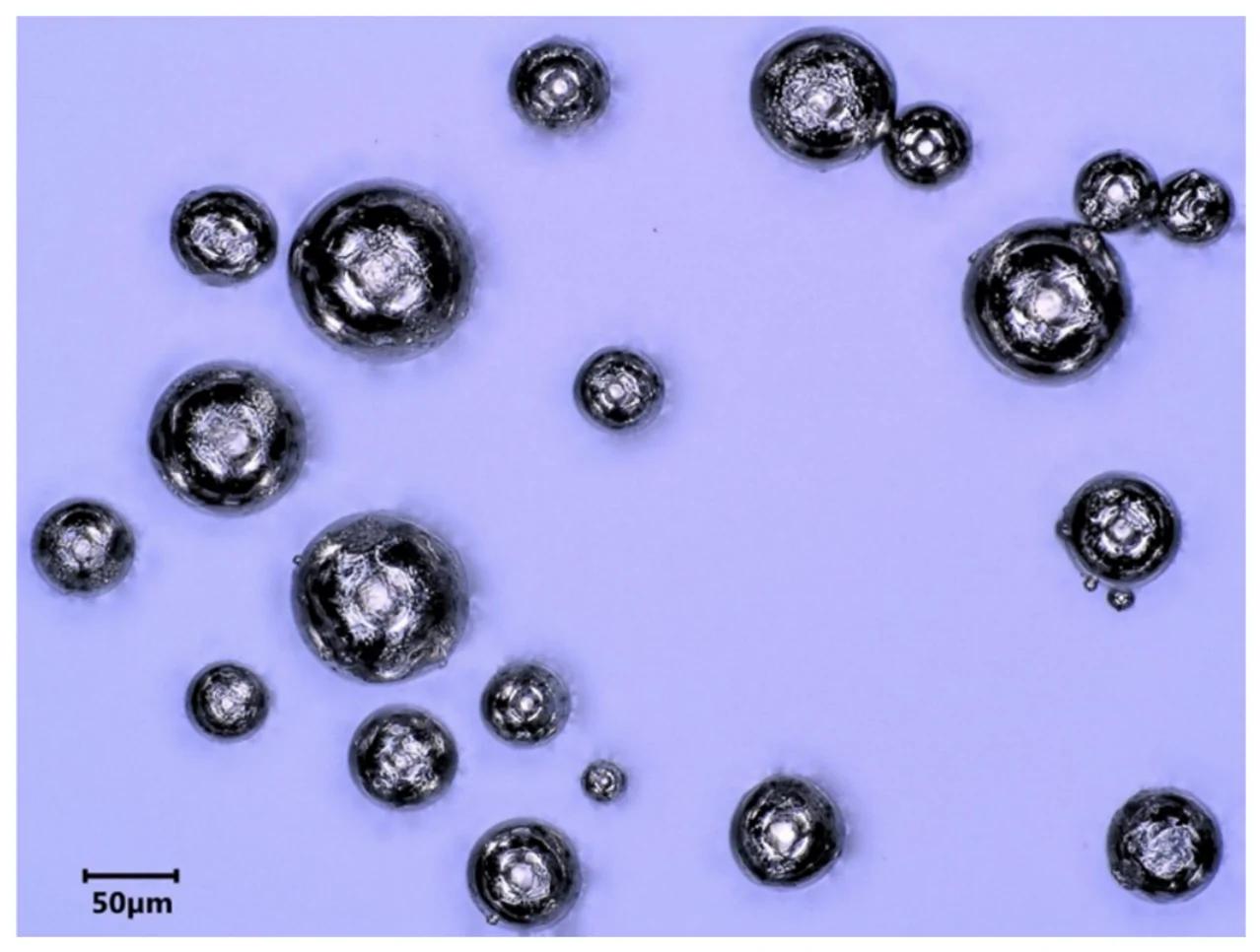
Ti-6Al-4V spherical powder used in this work.

**Figure 2:**
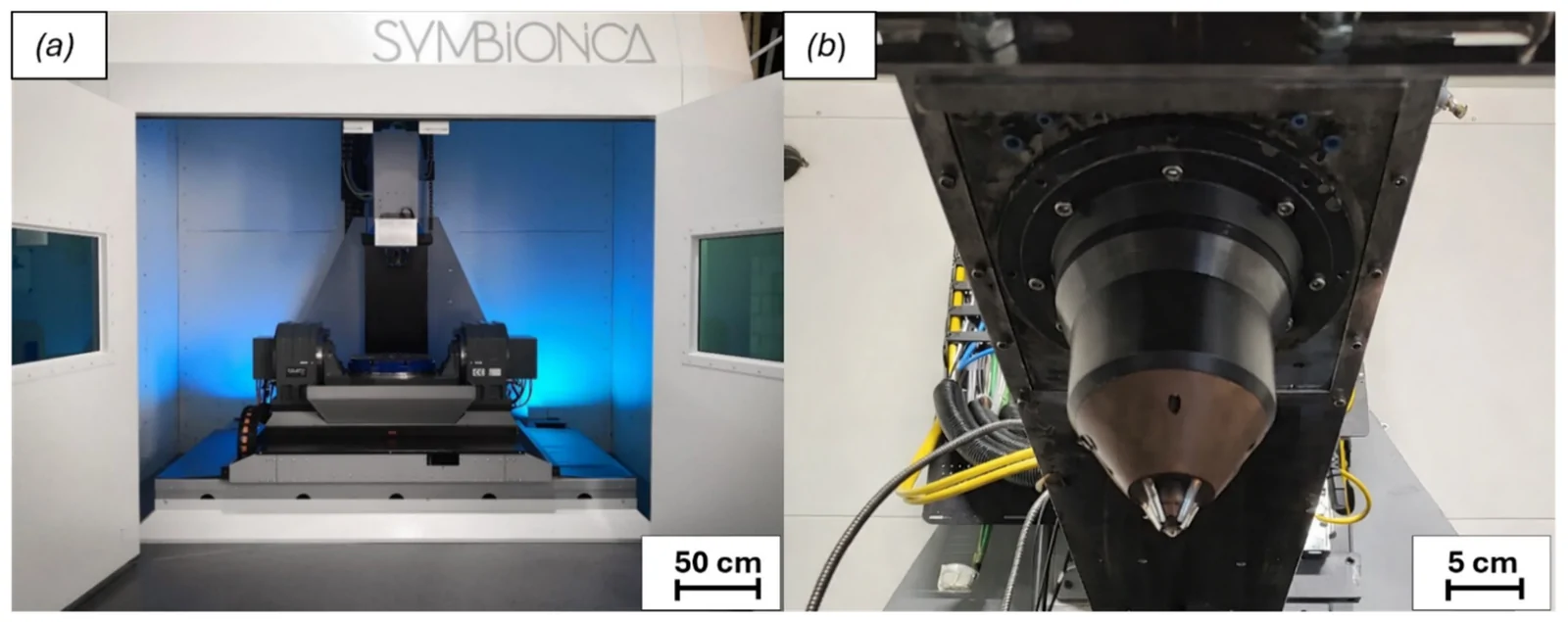
(**a**) Symbionica DED machine (**b**) Deposition head.

**Figure 3:**
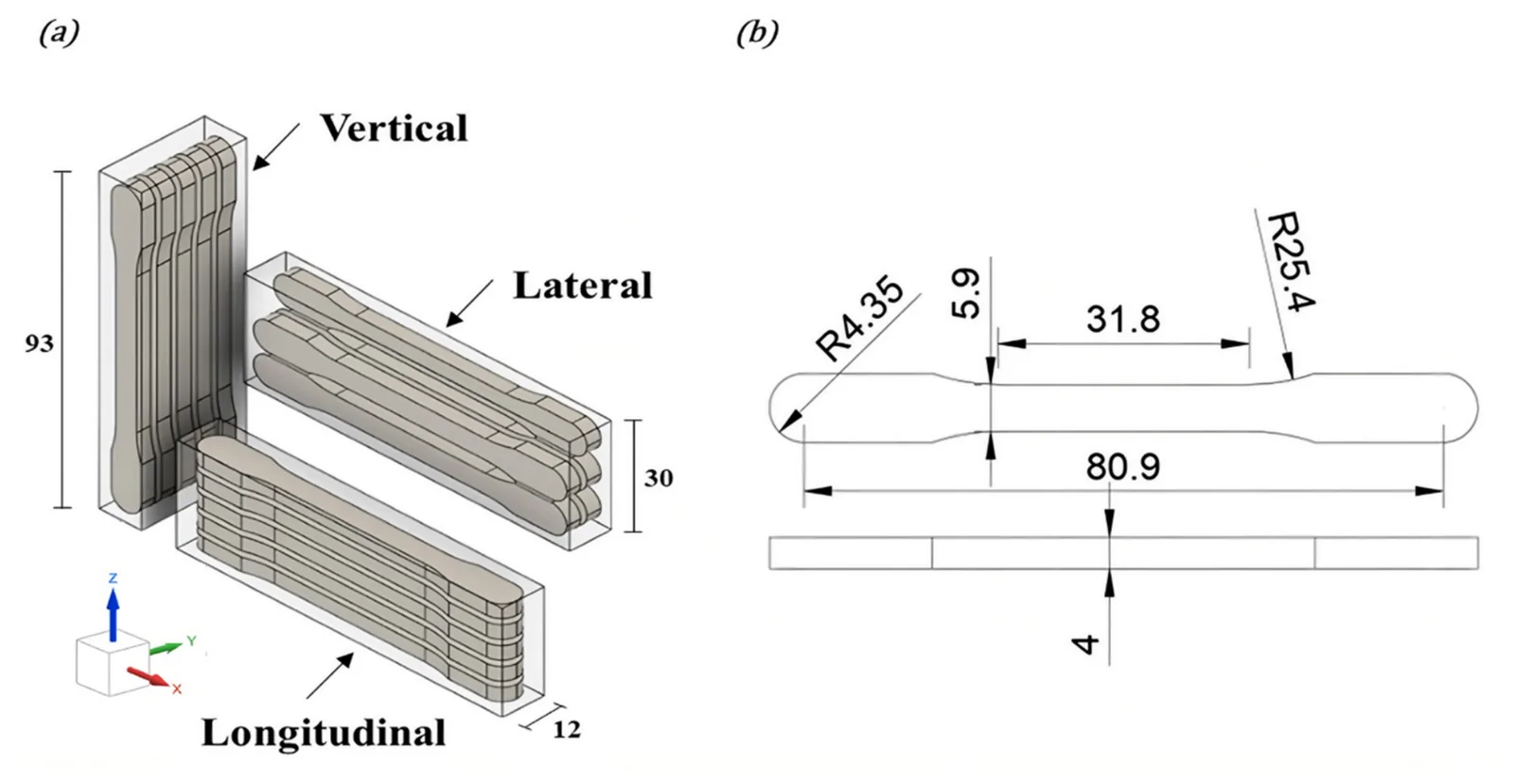
Scheme of (**a**) the method of tensile sample extraction at different build orientations; (**b**) Tensile Sample Geometry [mm].

**Figure 4:**
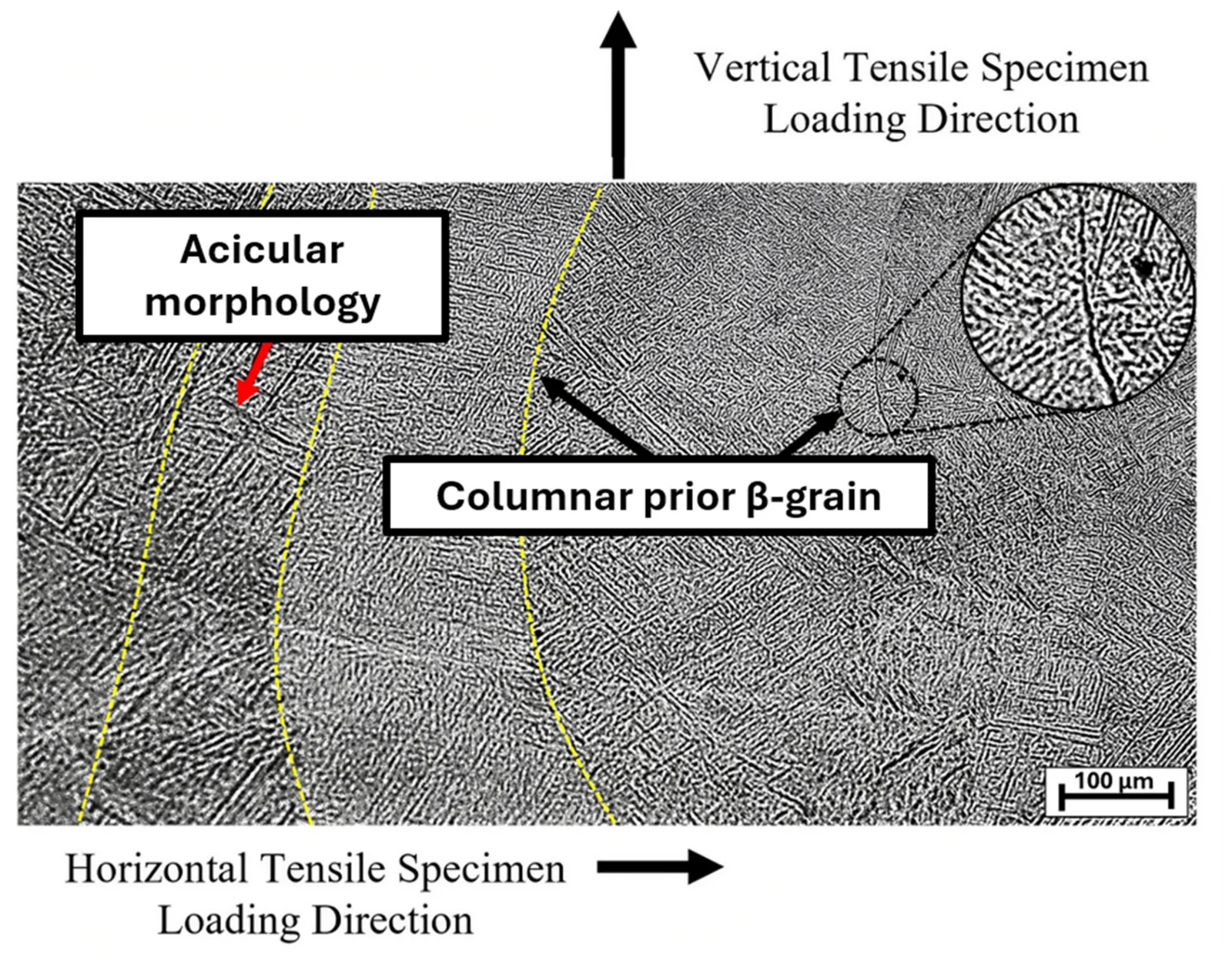
Optical micrograph of horizontally oriented tensile specimens (P4F9_Longitudinal), showing parallel elongated prior β-grains and a basket-weave interior structure. The loading direction concerning the microstructure is shown for both the horizontal and vertical build directions.

**Figure 5:**
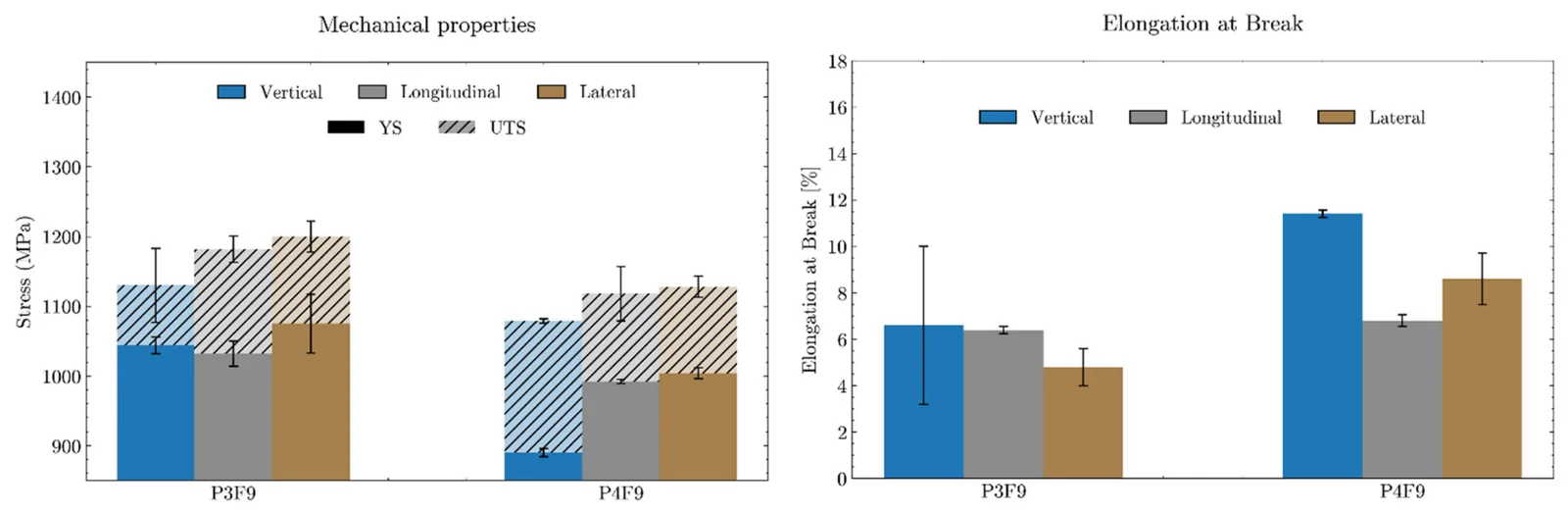
YS, UTS, and elongation at break of the Ti-6Al-4V samples in different directions.

**Figure 6:**
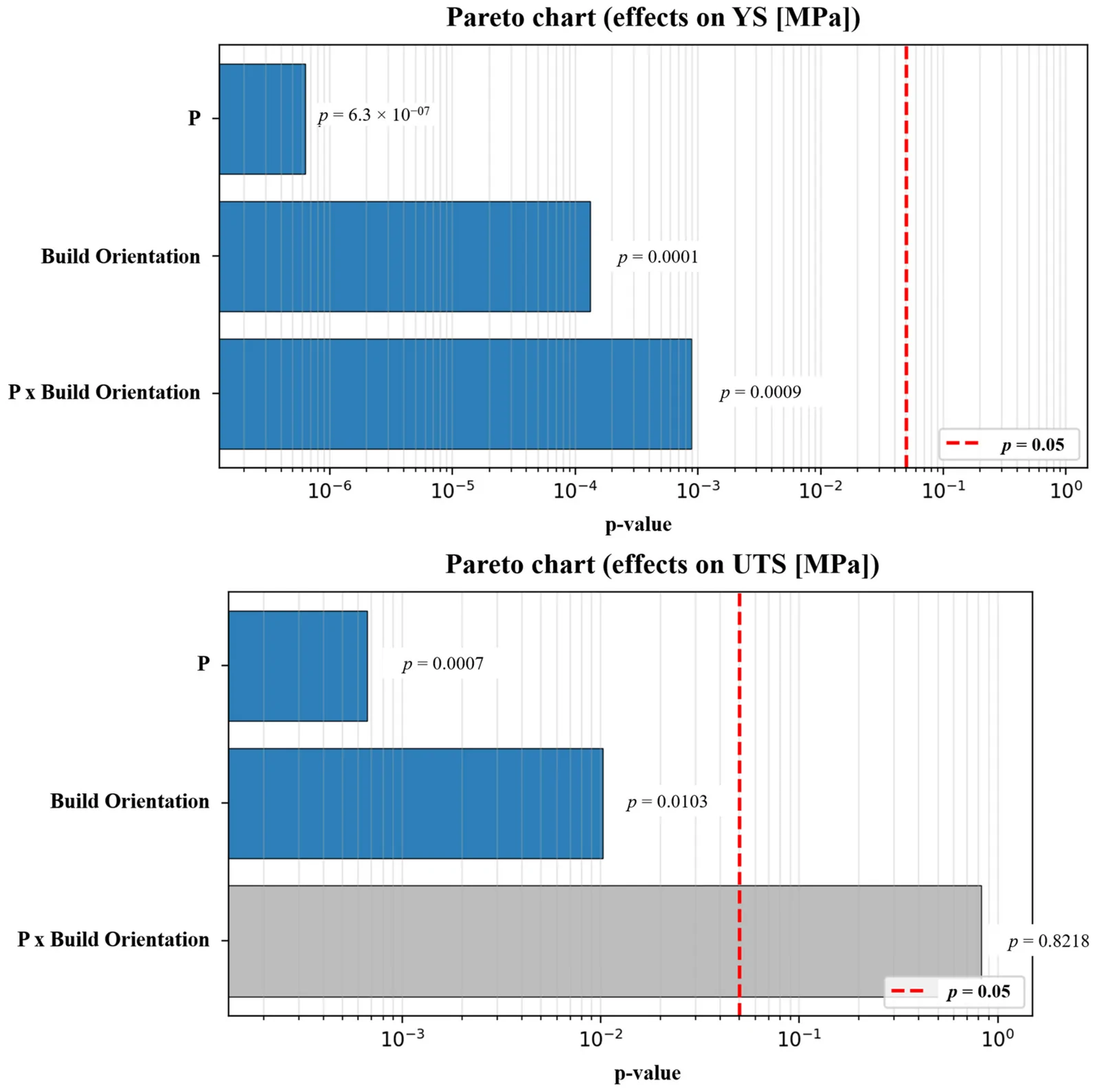

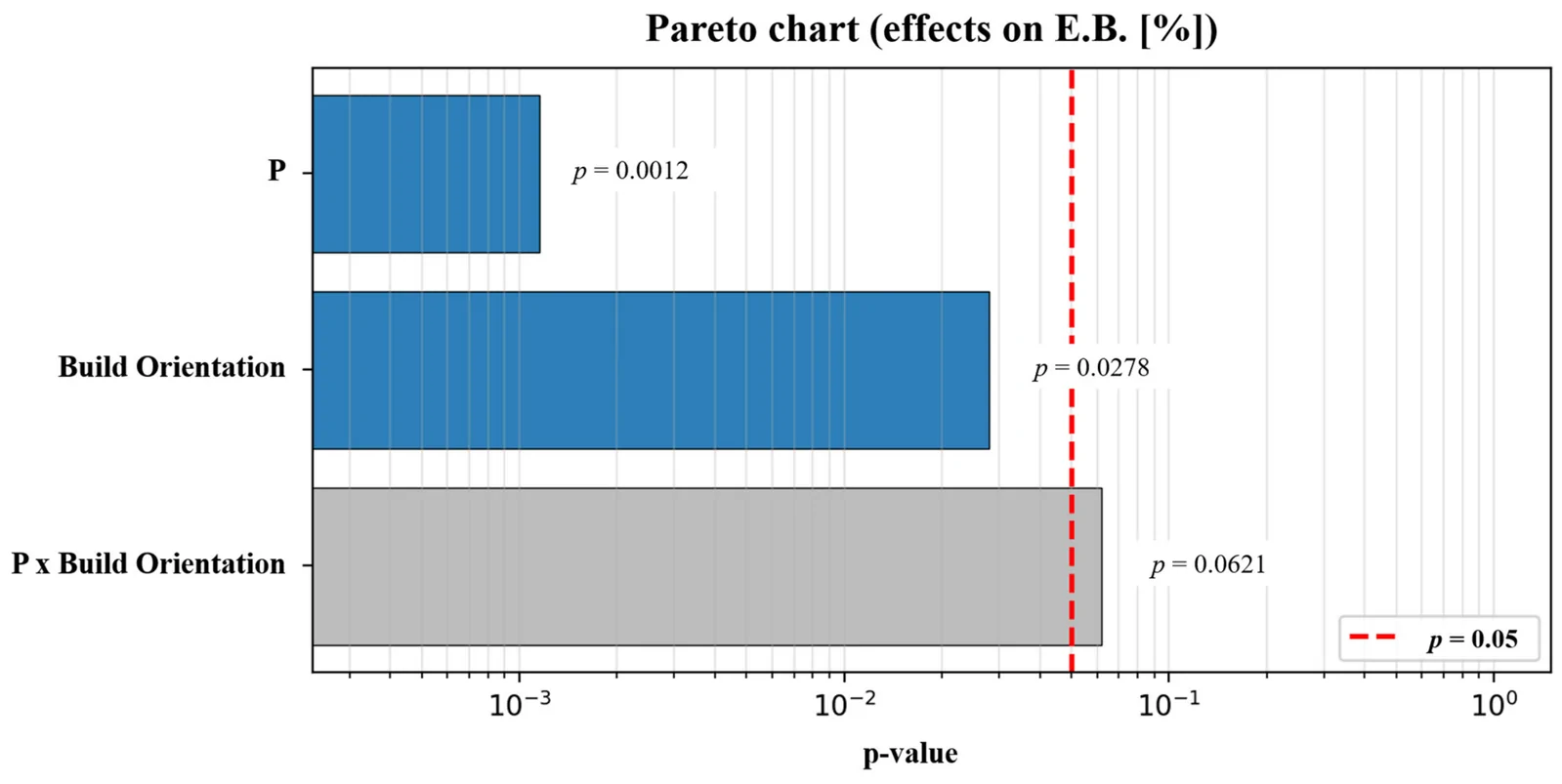
Pareto charts resulting from ANOVA for YS, UTS, and E.B.

**Figure 7:**
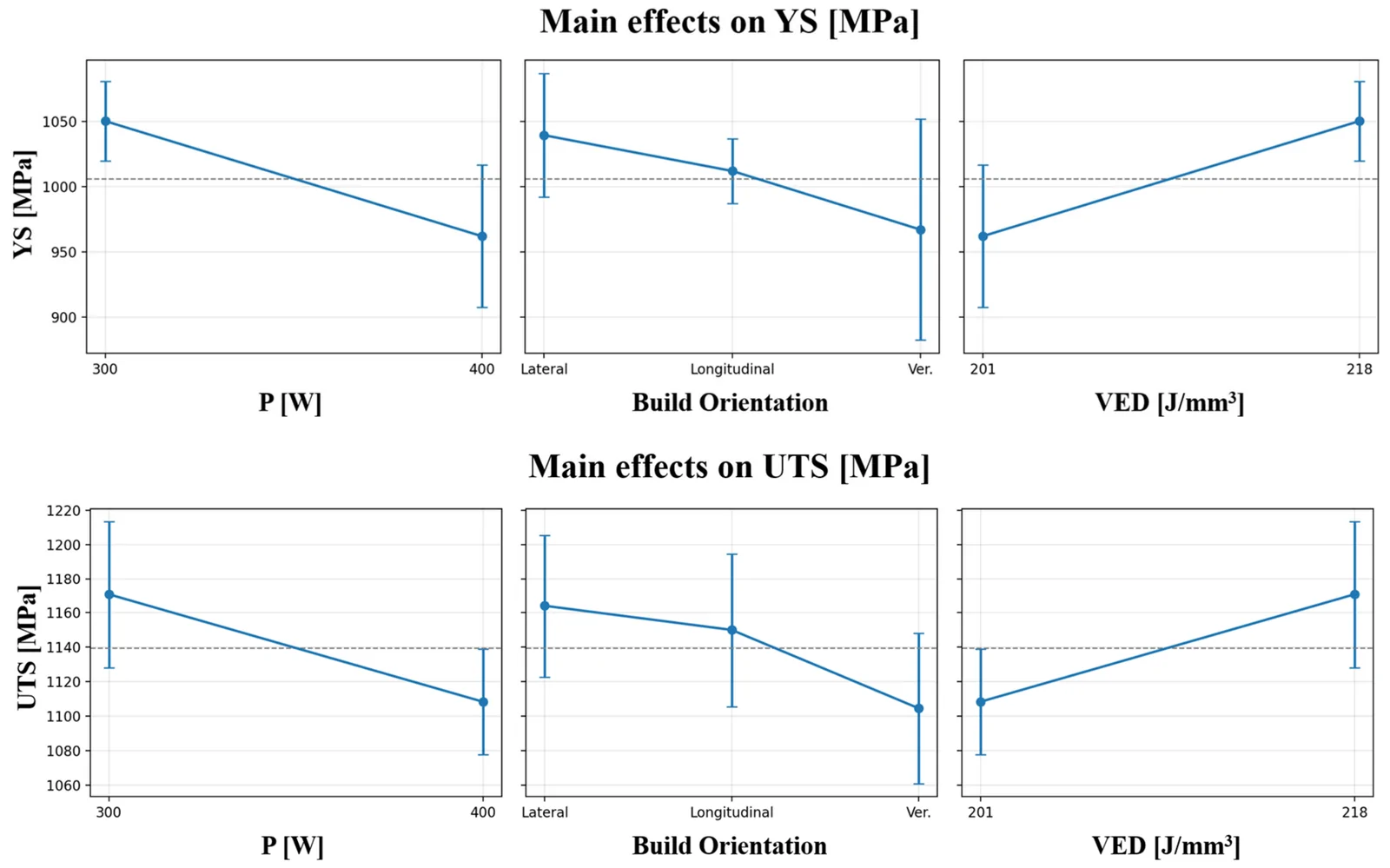

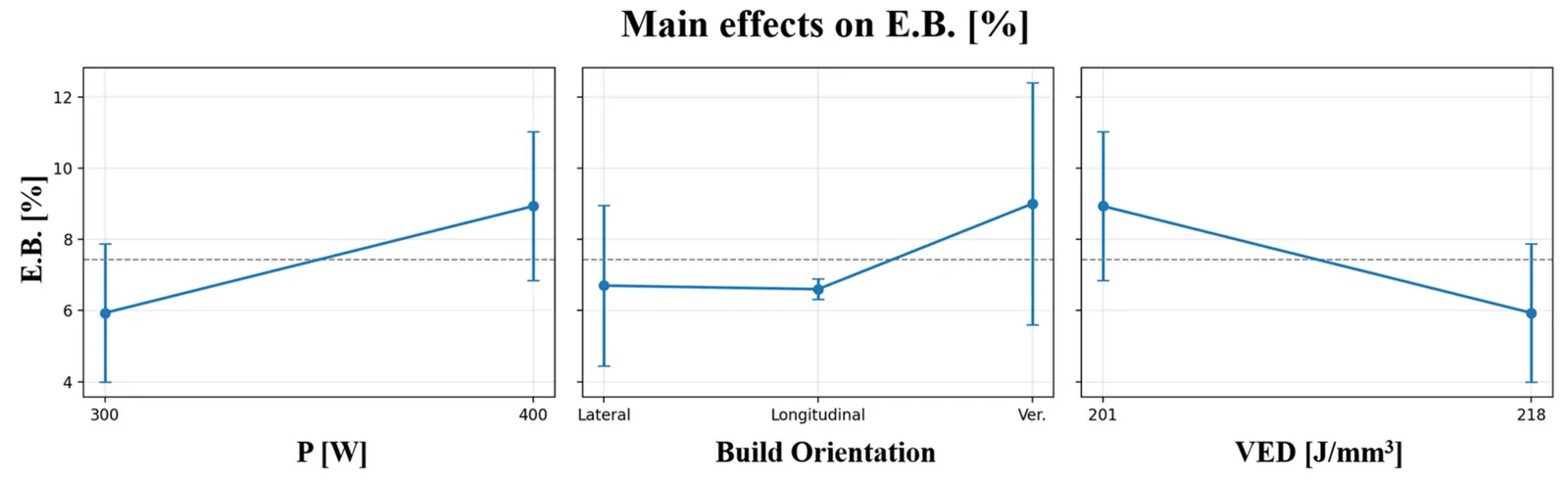
Main effect plots resulting from ANOVA for YS, UTS, and E.B.

**Figure 8:**
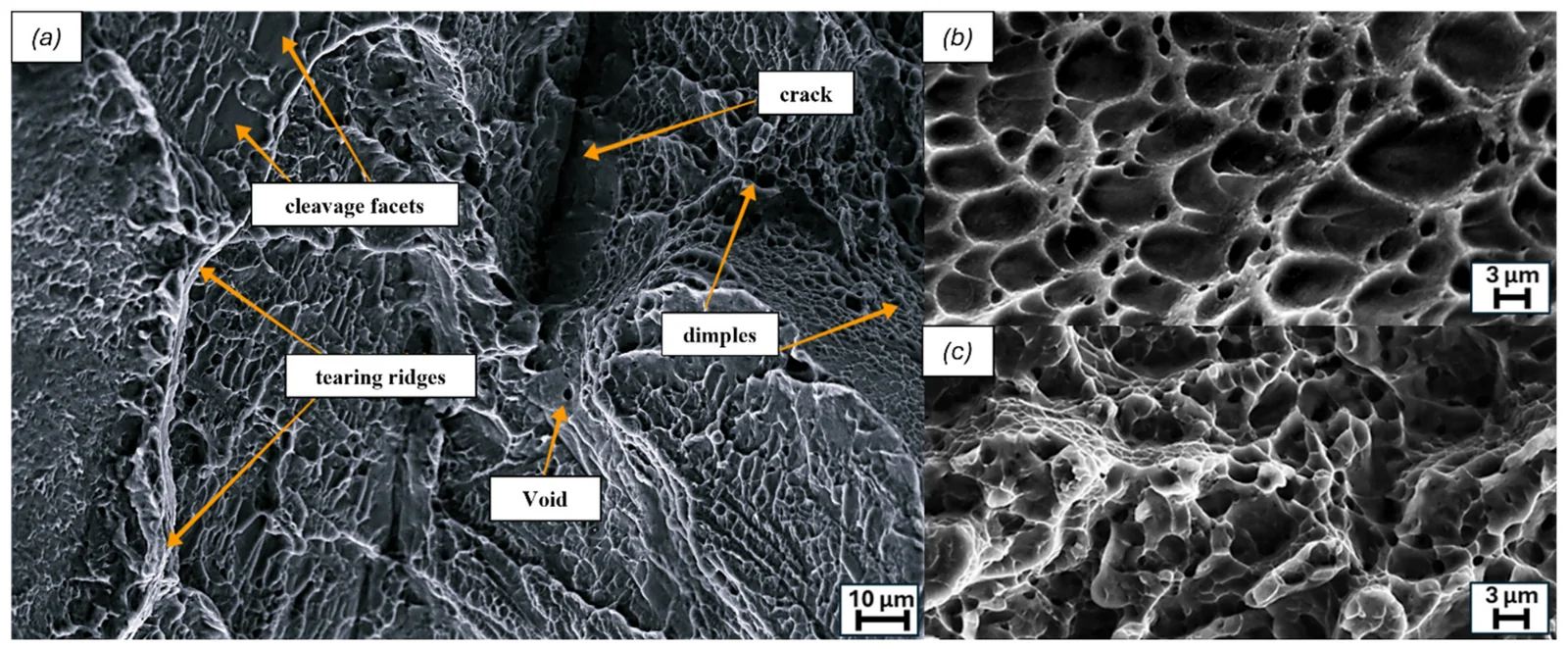
(**a**) SEM image of the tensile fracture surface of the P3F9_Longitudinal specimen, (**b**,**c**) Examples of microvoid coalescence in the fracture surfaces of specimens for different parameter sets and orientations. (**b**) P4F9_Vertical, and (**c**) P3F9_Longitudinal.

**Figure 9:**
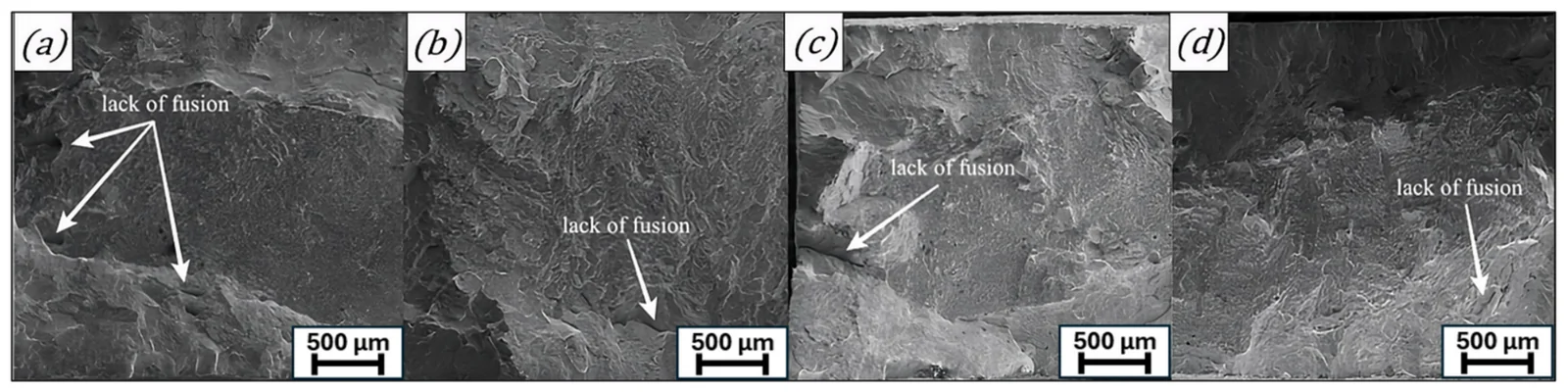
SEM images of the fracture surface of the tested samples: (**a**) P3F9_Longitudinal, (**b**) P3F9_Vertical, (**c**) P4F9_Longitudinal, and (**d**) P4F9_Vertical.

**Figure 10:**
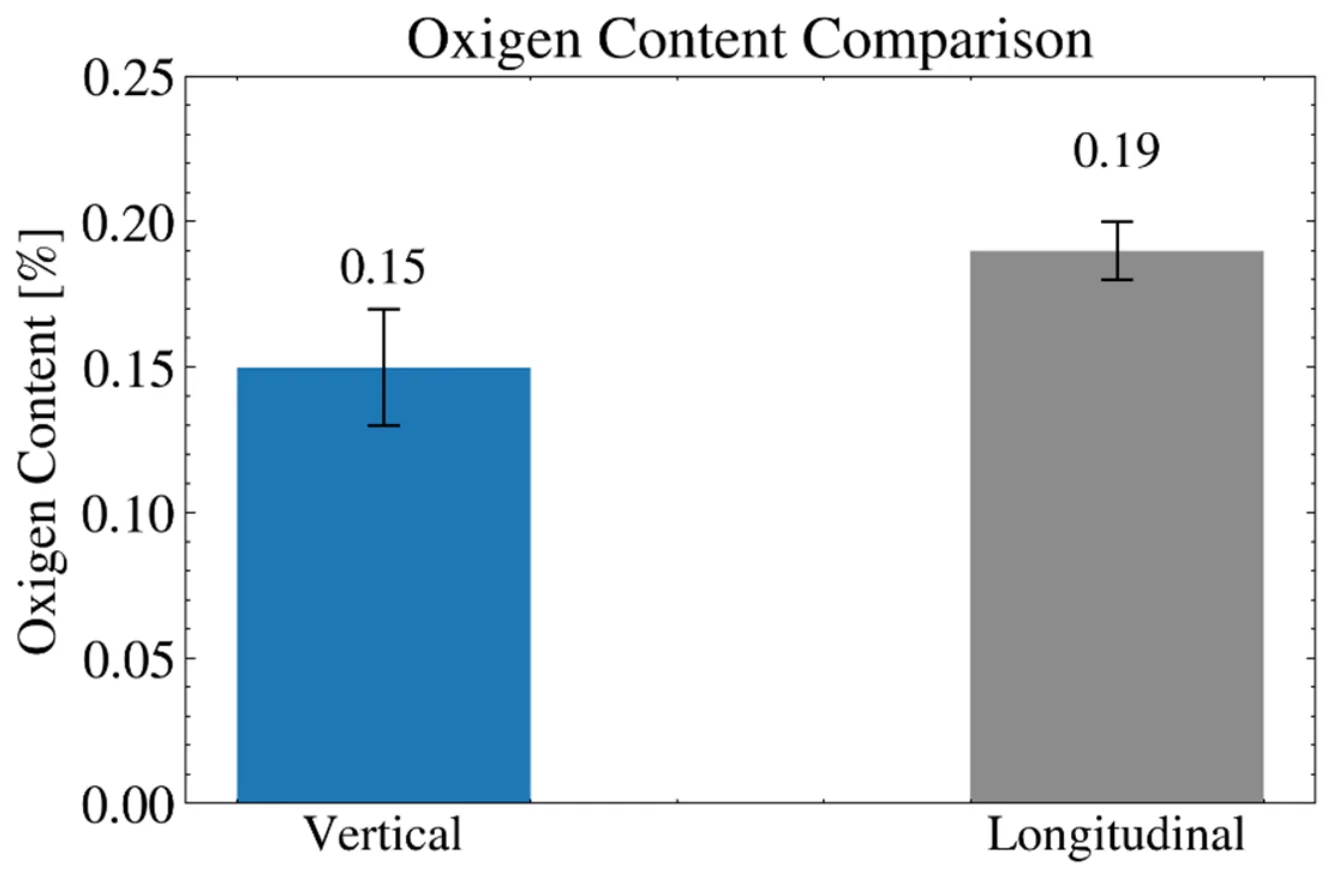
Oxygen content in the vertical and longitudinal Ti-6Al-4V specimens.

**Table 1:** Chemical composition of Ti-6Al-4V powder (wt.%).

Element	O	V	C	Fe	Al	Ti
Composition	≤0.20	3.9	0.01	0.18	6.49	Bal.

**Table 2:** Involved DED process parameters.

DED Process Parameters	P3F9	P4F9
Laser Power [W]	300	400
Axis feed rate [mm/min]	900	
Layer Thickness [µm]	191	240
Hatch distance [mm]	0.480	0.553
VED [J mm^−3^]	218	201
Substrate preheating [°C]	200	
Carrier flow [l/min]	6	
Shielding flow [l/min]	15	
Powder flow [g/min]	4.23	
Raster angle [°]	45	
Idle time between layers [s]	0	
Printing time [min]	118	93

**Table 3:** Resulting YS, UTS, and elongation at break.

Process Combinations	YS [MPa]	YS SD [MPa]	UTS [MPa]	UTS SD [MPa]	E.B. [%]	E.B. SD [%]
P3F9 Vertical	1044	+/− 12	1130	+/− 53	6.6	+/− 3.4
P3F9 Longitudinal	1032	+/− 18	1182	+/− 19	6.4	+/− 0.16
P3F9 Lateral	1075	+/− 42	1200	+/− 12	4.8	+/− 0.80
P4F9 Vertical	890	+/− 6	1079	+/− 3	11.4	+/− 0.16
P4F9 Longitudinal	992	+/− 3	1118	+/− 39	6.8	+/− 0.25
P4F9 Lateral	1004	+/− 8	1128	+/− 15	8.6	+/− 1.11

## Data Availability

The original contributions presented in this study are included in the article. Further inquiries can be directed to the corresponding author.
